# *Photorhabdus lux-*operon heat shock-like regulation

**DOI:** 10.1016/j.heliyon.2023.e14527

**Published:** 2023-03-11

**Authors:** V.V. Fomin, S.V. Bazhenov, O.V. Kononchuk, V.O. Matveeva, A.P. Zarubina, S.E. Spiridonov, I.V. Manukhov

**Affiliations:** a Laboratory of Molecular Genetics, Moscow Institute of Physics and Technology, Institutsky Lane 9, Dolgoprudny, Moscow Region, 141700, Russian Federation; b Laboratory of Microbiology, BIOTECH University, Volokolamskoe Highway 11, Moscow 125080, Russian Federation; c Biological Faculty, Lomonosov Moscow State University, Vorob’evy Gory, Moscow, 119992, Russian Federation; d Centre of Parasitology, A.N. Severtsov Institute of Ecology and Evolution, Russian Academy of Sciences, Leninskii Prospect, 33, Moscow, 119071, Russian Federation

**Keywords:** *P. temperata*, *Lux*-operon, Promoter, Heat shock

## Abstract

For decades, transcription of *Photorhabdus luminescens lux*-operon was considered being constitutive. Therefore, this *lux*-operon has been used for measurements in non-specific bacterial luminescent biosensors. Here, the expression of *Photorhabduslux*-operon under high temperature was studied. The expression was researched in the natural strain *Photorhabdus temperata* and in the heterologous system of *Escherichia coli*. *P. temperata* FV2201 bacterium was isolated from soil in the Moscow region (growth optimum 28 °C). We showed that its luminescence significantly increases when the temperature rises to 34 °C. The increase in luminescence is associated with an increase in the transcription of *luxCDABE* genes, which was confirmed by RT-PCR. The promoter of the *lux*-operon of the related bacterium *P. luminescens* ZM1 from the forests of Moldova, being cloned in the heterologous system of *E. coli*, is activated when the temperature rises from room temperature to 42 °C. When heat shock is caused by ethanol addition, transcription of *lux*-operon increases only in the natural strain of *P. temperata*, but not in the heterologous system of *E. coli* cells. In addition, the activation of the *lux*-operon of *P. luminescens* persists in *E. coli* strains deficient in both the *rpoH* and *rpoE* genes. These results indicate the presence of sigma 32 and sigma 24 independent heat-shock-like mechanism of regulation of the *lux*-operon of *P. luminescens* in the heterologous *E. coli* system.

## 1 Introduction

Enterobacteria of the genus *Photorhabdus* (formerly *Xenorhabdus*) mostly are terrestrial bioluminescent bacteria living in the intestines of entomopathogenic nematodes *Heterorhabditis* [1]. Previously *Photorhabdus* isolates were divided into 3 new species: *P. luminescens*, *P. temperata* and *Photorhabdus asymbiotica* [1]. *P. luminescens* (containing the type strain) and *P. temperata* are symbiotic representatives with max growth temperatures 35–39 °C and 33–35 °C respectively [1]. *P. asymbiotica* isolates have been collected from a human host [1,2].

*P. luminescens* cells can switch between two forms: the pathogenic to insects P-form and the mutualistic M-form, which appears in the intestines of nematodes. It was shown in Refs. [3,4] that the transition from one form to another is controlled by the orientation of the *mad*-operon promoter. In the P-form, these bacteria have a more pronounced luminescence compared to the M-form, release toxins to kill insects, antibiotics to fight other microorganisms, as well as biologically active substances for the successful reproduction of itself and the host nematodes [3,5]. Various substances that can act as antibiotics against both Gram-positive and Gram-negative bacteria are described in the review [6]. For example, novel antibiotic versus Gram-negative bacteria named Darobactin have been found from screening of *Photorhabdus* isolates [7]. Also, the possibility of application of *Photorhabdus* toxins against Colorado potato beetle and mosquitoes has been discussed [8,9].

Many bioluminescent bacteria are characterized by induction of *lux*-operon expression depending on cell density (quorum sensing) and environmental conditions [[10], [11], [12]]. Also, commonly the glow of bacteria depends on various factors: cAMP concentration, iron level, oxygen concentration, osmolarity, etc [13]. However, for some representatives of the luminescent microflora, the mechanisms of luminosity regulation have not been identified. In particular, it is believed that luminescence is constitutive among representatives of the genera *Photorhabdus* and *Photobacterium* [[14], [15], [16], [17], [18], [19], [20]].

The temperature optimum of various bacterial luciferases basically corresponds to the living conditions of the corresponding bacteria and ranges from 15 to 39 °C [2,11,21,22]. The thermal stability of luciferases *in vivo* is affected by the rate of refolding, which occurs with the participation of chaperones DnaKJE, TF, ClpA, ClpB, IbpAB [17,18,[23], [24], [25]].

Under heat shock conditions, the synthesis of many chaperones and proteases is activated. In *E. coli*, the main inducer of transcriptional synthesis of heat shock proteins is the σ^32^ factor (RpoH) of RNA-polymerase [26]. The tenfold increase in the heat shock proteins synthesis is observed when temperature rises from 30 to 42 °C [27]. This effect is achieved due to the complex regulation of activity and synthesis of σ^32^ [26]. In addition to σ^32^, *E. coli* cells synthesize the σ^E^ factor, the activation of which is controlled by improperly folded periplasmic proteins (heat shock from 30 to 46 °C) [28].

Cells carrying *P. luminescens lux*-operon under control of its own promoter are often applied as non-specific biosensor for integral toxicity assessment [19,20,29,30]. Ability of native *lux*-operon to be induced under some conditions can interfere with such investigations. Here, we determined the influence of temperature conditions on the expression of *lux*-genes under native promoter in *E. coli* cells, carrying *lux*-operon from *P. luminescens* ZM1, and in the natural strain *P. temperata*. The expression of the *lux*-operon in cells was compared by unit luminescence at various temperatures ranging from room temperature to 42 °C, and at different concentrations of ethanol. To prove *lux*-operon promoter activation, transcription intensity of *Photorhabdus lux*-operons was analyzed by RT-PCR technique.

## 2 Materials and methods

### 2.1 Bacterial strains and plasmids

Bacteria and plasmids are listed in Table 1 and Table 2, respectively. Construction of vectors is described in the supplementary materials. Primers used in the study are given in Table S1 (see Supplementary file).

**Table 1:** Bacterial strains of *E. coli* and *P. temperata*.

Bacterium	Description	Source
*E. coli* MC1061	F–, *Δ(araA-leu)7697*, *[araD139]*_*B/r*_, *Δ(codB-lacI)3*, *galK16*, *galE15*(GalS), *λ*^*-*^, *e14-*, *mcrA0*, *relA1*, *rpsL150*(strR), *spoT1*, *mcrB1*, *hsdR2*	VKPM collection
*E. coli* MG1655	F–, *λ*^*–*^, *ilvG–*, *rfb-50*, *rph-1*	VKPM collection
*E. coli* MG1655 *ΔrpoE*	F–, *λ*^*–*^, *ilvG–*, *rfb-50*, *rph-1, ΔrpoE::*Cm	Kindly provided by Zakataeva N.P., Ajinomoto-Genetika Research Institute
*E. coli* K165	F–, *lacZ53*(Am), *phoA5*(Am), *λ*^*-*^, *tyrT47*(OS), *tyrT91*(ts, AS), *trp-48* (Am), *relA1*, *rpsL150*(strR), *malT66*(Am, λ^R^), *rpoH601*(Am), *spoT1*	VKPM collection
*P. temperata* FV2201	Natural strain isolated from infected *Galleria mellonella*	This study

**Table 2:** Plasmids used in the study

Plasmid	Description	Source
pXen7	Plasmid based on pUC18 with *lux*-operon *P. luminescens* ZM1 under the control of its own promoter	[31]
pDEW201	The replication origin of pBR322; promoterless operon *P. luminescens luxCDABE* genes; Ap^r^	[32]
pGrpE-lux	pDEW201-based plasmid with insertion of the *GrpE* gene promoter	[19]
pXenP	Plasmid based on pDEW201 with the insertion of a promoter site of the *lux*-operon *P. luminescens* ZM1.	This study
pDlac	Plasmid based on pDEW201 with the insertion of a P_*lac*_ promoter	This study

### 2.2 Isolation of a natural strain of *P. temperata*

**Fig. 1:**
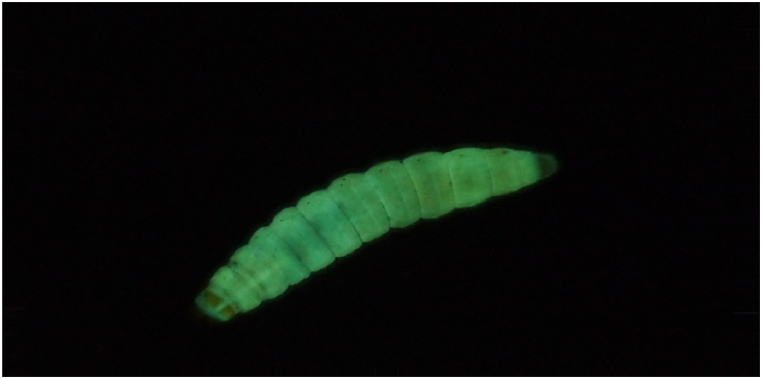
*G. mellonella* infected with *P. temperata*. Shooting with a camera in the dark.

Soil samples weighing 2–3 kg were collected in sandy soil under young pine plantings near the territory of the Serebryany Bor Park (55.7807, 37.4281). Soil samples were delivered to the laboratory, where it was distributed in 500 mL plastic containers, filling it to the brim so that the lid pressing the soil did not leave empty spaces inside. 5 caterpillars of wax moth (large wax fireworm *G. mellonella*) were added to each container. After 3–4 days, the condition of the insects was checked, and if dead ones were found, evenly colored in red, dark gray or crimson, then such dead insects were placed singly on filter paper in a Petri dish with a diameter of 40 mm, which was wrapped with Parafilm (Parafilm ®) to prevent drying. Daily observations of the condition of the deceased insect were carried out, and at the first signs of the appearance of numerous invasive larvae on its surface (length about 700 μm), the lower part of a small Petri dish was transferred to a large cup filled with 1–2 mm of standing tap water. Upon the release of the formed larvae into the water, the suspension was transferred to plastic flattened flasks with screw caps (Falcon or similar). In this form, they were stored at 4–6 °C. The resulting larvae infected with bacteria had the ability to bioluminesce (Fig. 1).

The internal contents of the infected larva were dissolved in a liquid LB medium for liquid seeding on an agarized medium. As a result, separate luminous bacterial colonies were obtained, which were later used in the work. Sequencing of the 16S ribosomal gene using primers 16S_Ph_unidir and 16S_Ph_univer (Table S1) and comparison of the obtained sequence in the NCBI database by the BLAST method showed kinship to *P. temperata*.

### 2.3 Cultivation of bacterial cells

*E. coli* and *P. temperata* grew in LB medium (1% trypton, 0.5% yeast extract, 1% NaCl) with constant aeration at 200 rpm at the temperature specified in the experiment. The solid medium for Petri dishes was prepared with the addition of 1.5% agar.

The following concentrations of antibiotics (μg/ml) were used for selective media: ampicillin – 100, chloramphenicol – 25, streptomycin – 25.

The optical density was measured with a KFK-2 photometer (ZOMP, Russia).

### 2.4 DNA manipulation

Plasmid DNA was isolated with the use of GeneJET Plasmid Miniprep Kit (Thermo Scientific, Waltham, MA, USA). Cell transformation with hybrid plasmids, agarose gel electrophoresis, and isolation of DNA fragments from agarose gel were performed according to the Sambrook manual [33]. Restriction and ligation reactions were carried out using enzymes from Promega (Madison, WI, USA).

### 2.5 Luminescence measurement

The measurement of the luminescence level was conducted as described before [12]. In brief, a cell suspension was transferred into 200 μL tubes, then the tube was placed in a Biotox-7BM (BioPhysTech, Russia) luminometer to measure the total luminescence level (in relative units) at room temperature (23 °C). Measurements were conducted after 5 min of incubation at 23 °C to cool samples to standard conditions. Highly luminous samples were 10- or 100-fold diluted to match the dynamic range of the luminometer.

### 2.6 Design of experiments to evaluate luminescence dependence on temperature

Luminous bacterial cells were grown in liquid LB at 23 °C (28 °C in the measurements with ethanol in the medium) and 200 rpm to an optical density of OD_600_ ∼ 0.1. Then each investigated cell suspension was divided into equal portions, which were further incubated at different temperatures. At the end of incubation or at specific moments, the luminescence and optical density of cell cultures were measured.

To take into account the difference in growth rate of *E. coli* and *P. temperata* cells at different temperatures the unit luminescence was calculated for each point as follows:

(1) $$UL=\frac{Lum}{OD}\times d$$

where UL - unit luminescence of sample, Lum - total luminescence produced by the sample, OD - optical density of cell culture in the sample, *d* – dilution factor.

To unify all experimental replicates coefficient of induction for each temperature was calculated as follows:

(2) $$K\left(T\right)=\frac{UL\left(T\right)}{UL\left(T_{0}\right)}$$

Where K(T) - coefficient of induction of luminescence, T_0_ - the lowest temperature in the experiment (23 or 28 °C).

When ethanol was used to stimulate heat shock, the final concentration of ethanol in the medium was varied instead of temperature, and the luminescence of the sample without spirit was used in equation (2) in denominator.

### 2.7 RNA extraction and RT-PCR

Bacterial cells of *E. coli* MG1655 pXen7 and *P. temperata* grew at 28 °C and 200 rpm to an optical density of OD_600_ ∼ 0.1. Then the cell suspension was divided into samples at 28 and 34 °C and incubated for 3 h. After the time elapsed, the luminescence and optical density were measured. Next, the equivalent of 3 OD∙ml of bacterial cells (volume depends on optical density) was harvested and total RNA was extracted according to BioRad Aurum Total RNA Kit manual.

Reverse transcription polymerase chain reaction (RT-PCR) was performed to estimate relative transcriptional activity of *lux*-operons of *P. temperata* and *E. coli* MG1655 pXen7 cells when they had been grown at higher temperature conditions. It was done for both samples by evaluating contents of target gene of *lux*-operons - *luxC* normalized to housekeeping 16S ribosomal gene from extracted total RNA. To show changes in transcription, ΔΔ*C*_T_ [34] was calculated:

(3) $$\Delta \Delta C_{T}=\left(C_{T,{luxC}_{34{}^{\circ}C}}-C_{T,{16s}_{34{}^{\circ}C}}\right)-\left(C_{T,{luxC}_{28{}^{\circ}C}}-C_{T,{16s}_{28{}^{\circ}C}}\right)$$

Where *C*_T_ is a threshold cycle for exact probe, it was calculated using software Rotor-Gene 6000 (Corbett Research, Australia).

RT-PCR was done using the OneTube RT-PCR SYBR kit (Eurogen, Moscow, Russia) according to the procedure specified by the manufacturer in Rotor-Gene Q amplifier. 0.5 ng of extracted total RNA was added to the finished mixture, the concentration of which was measured with a NanoPhotometer ® P 330 spectrophotometer. Primers Ph_DluxC and Ph_RluxC (Table S1) flanked a region of ∼400 nucleotides of the target *luxC* gene. The fragment of ∼400 nucleotides of conservatively expressed 16S ribosomal gene was amplified by universal primers 16SuniD and 16SuniRmidi (Table S1). Amplification mode: 50 °C - 15 min, 95 °C - 1 min, 45 cycles: 95 °C - 15 s; 55 °C - 20 s; 72 °C - 25 s. Obtaining melting curves: 50 °C - 1 min, from 50 °C to 94 °C in increments of 1 °C–5 s.

Efficiency of amplification in the experiment wasn’t measured however should be almost the same for primers with close annealing temperatures and similar length of PCR products. Therefore, the exact relevant amount of *luxC* at temperature 34 °C with respect to 28 °C is not calculated.

### 2.8 Statistics

All experiments with luminescence measurements with *E. coli* and *P. temperata* cell cultures were performed in triplicate. RNA extraction and RT-PCR analysis were done in duplicate. The error of induction coefficient and ΔΔ*C*_T_ were calculated by the formula of standard deviation on the basis of three or two replicates, respectively. Its value on the graphs is represented by error bars.

## 3 Results

### 3.1 Activation of the *lux*-operon in the heterologous *E. coli* system

**Fig. 2:**
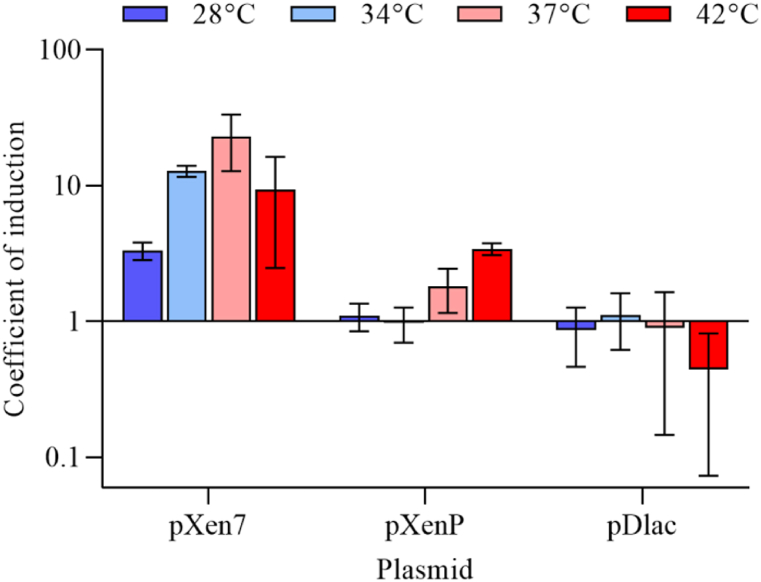
Coefficients of induction of luminescence of *E. coli* MG1655 cells with pXen7, pXenP, and pDlac plasmids after 14–16 h at 28, 34, 37, and 42 °C without shaking. Data points and error bars correspond to means and standard deviations determined in three independent experiments.

To investigate the temperature-dependent activation of expression of luminescence-encoding genes under the control of the promoter of *lux*-operon from *Photorhabdus* bacteria, *E. coli* MG1655 cells transformed with pXen7, pXenP, and pDlac plasmids grew for 14–16 h (overnight) at 23, 28, 34, 37, and 42 °C without shaking. Then the luminescence intensity was measured, as well as the optical density. On the histogram (Fig. 2) the coefficients of induction (3) are given.

As shown on the histogram (Fig. 2), *E. coli* cells with pXen7 and pXenP plasmids containing the *lux*-operon promoter *P. luminescens* ZM1 demonstrate an increase in the level of luminescence with increasing of temperature, in contrast to cells with pDlac plasmid, in which *luxCDABE* genes are under the control of non-activated heat shock, constitutive, under these conditions, the P_*lac*_ promoter.

**Fig. 3:**
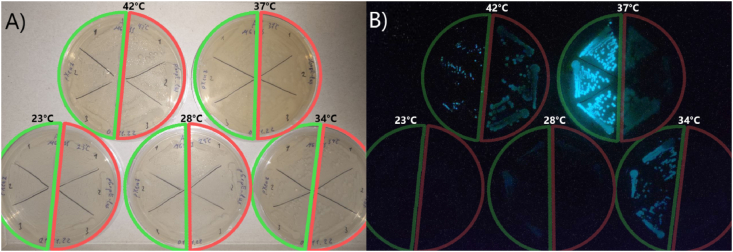
The pictures of plates with cells of *E. coli* MG1655 pXen7 and *E. coli* MG1655 pGrpE-lux after incubation overnight at 23, 28, 34, 37, and 42 °C. The A) and B) pictures are taken in the light and in the dark, respectively. Segments with cultures carrying pXen7 are outlined in green; pGrpE-lux ― red. Temperature of overnight incubation of every plate is indicated in the picture.

Induction of *lux*-genes expression under heat shock conditions can be observed clearly in Fig. 3, where *E. coli* MG1655 pXen7 and specific biosensor for heat shock *E. coli* MG1655 pGrpE-lux were grown overnight on plates at 23, 28, 34, 37, and 42 °C.

### 3.2 Induction of luminescence of *P. temperata* strain

For experiments with *lux*-operon in native host, we conducted a search for luminescent bacteria in the Moscow region and succeeded to isolate *P. temperata* FV2201 strain. It turned out that overnight incubation at 34 °C is very poorly for growth of *P. temperata* cells. Moreover, when incubated for 3 h at 37 °C, the unit luminosity is lower than at 34 °C, and at 40 °C they stop to luminesce at all. It is understandable, since the max growth temperature for *P. temperata* is 33–35 °C.

**Fig. 4:**
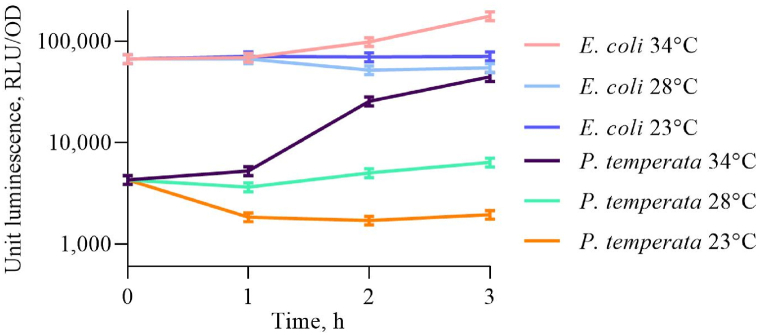
Luminescence change kinetics of *P. temperata* FV2201 and *E. coli* MG1655 pXen7 cells after split and incubation at 23, 28, and 34 °C. Unit luminescence was calculated according to (1). The initial value corresponds to the moment of separation into equal samples when OD_600_ ∼ 0.1 is reached.

*P. temperata* FV2201 and *E. coli* MG1655 pXen7 cells suspensions after incubation were divided into equal samples, which continued growth at 23, 28, and 34 °C. Fig. 4 shows characteristic dependence of the specific luminescence (1) on the incubation time at the indicated temperatures. Additional experimental data could be found in the Supplementary materials (Table S2).

It can be observed that at 34 °C both *E. coli* MG1655 pXen7 and *P. temperata* cell suspension have more intensive expression of *lux*-genes with regard to lower temperatures 23 and 28 °C.

### 3.3 Induction of heat shock by ethanol

**Fig. 5:**
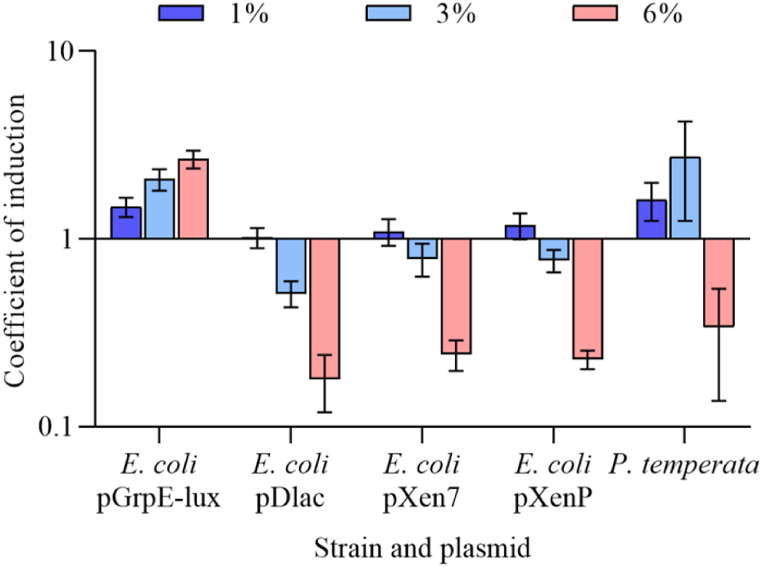
Induction coefficients of luminescence of *E. coli* MG1655 cells transformed with pGrpE-lux, pDlac, pXen7, or pXenP and *P. temperata* FV2201 cells after 2 h at room temperature, when ethanol was added to the medium at final concentrations of 1, 3, and 6%. Means and standard deviations were determined in three independent experiments.

To test the protein-mediated response of *lux*-operons in the presence of ethanol, the following measures were conducted. *E. coli* MG1655 cells carrying plasmids pGrpE-lux, pDlac, pXen7 or pXenP, and *P. temperata* cell suspensions were divided into 200 μL portions and supplemented with ethanol at final concentrations of 0, 1, 3, and 6%. Resulting samples were incubated at room temperature for 2 h. The data obtained is shown in Fig. 5.

On the graph, the activation of luciferase synthesis in the presence of ethanol in the *E. coli* MG1655 pDlac cells does not occur. The specific biosensor for heat shock of *E. coli* MG1655 pGrpE-lux is reliably activated by all the ethanol concentrations studied. For cells with pXen7 and pXenP, the activation of luminescence at 1% ethanol is low and unreliable. Higher concentrations reduce luminescence due to toxicity. On the contrary, the synthesis of *lux*-genes of the *P. temperata* strain significantly increases at ethanol concentrations of 1 and 3%. A higher concentration of 6% reduces luminescence due to general toxicity.

### 3.4 Analysis of transcriptional activity

To assess changes in the transcription of *lux*-operon promoter, *E. coli* MG1655 pXen7 and *P. temperata* cells were incubated at 28 and 34 °C for 3 h, followed by total RNA extraction and RT-PCR. At these temperatures, there is still a small difference in the rate of cell growth, but a significant increase in luminescence is observed. The luminescence level and optical density of the samples were also measured to control the induction of expression. *luxC* and 16S rRNA genes fragments were amplified by RT-PCR and unit content of *lux*-operon mRNA was estimated.

The results of the calculation of ΔΔ*C*_T_ (3) are as follows: ΔΔ*C*_T_ are −3.54 ± 1.47 for *P. temperata* FV2201 and -1.53 ± 0.74 for *E. coli* MG1655 pXen7 cells. Comparison of unit luminescence (1) for these samples gave the following coefficients of induction: 12.5 ± 7.3 and 6.4 ± 2.4, respectively.

According to the data, both *luxC* gene transcription and unit luminescence increase with an increase in the incubation temperature ranging from 28 to 34 °C. The values obtained are almost the same for *P. temperata* cells and the *lux*-operon of *P. luminescens* in a heterologous *E. coli* cell system. The bacterial luminescence is expected to be proportional to the square of the activity of *luxAB* transcription [35], thus taking into account that PCR effectiveness was lower, then doubling per cycle, the activation coefficients correspond to the values of ΔΔ*C*_T_. This allows us to conclude that the luminescence enhancement described in this paper with an increase in temperature is due to the regulation of the *lux*-operon promoter. Moreover, this regulation persists even when the *lux*-operon is transferred to a heterologous system of an unrelated bacterial species.

### 3.5 Influence of σ^32^ and σ^24^ on activation of the *lux*-operon promoter

**Fig. 6:**
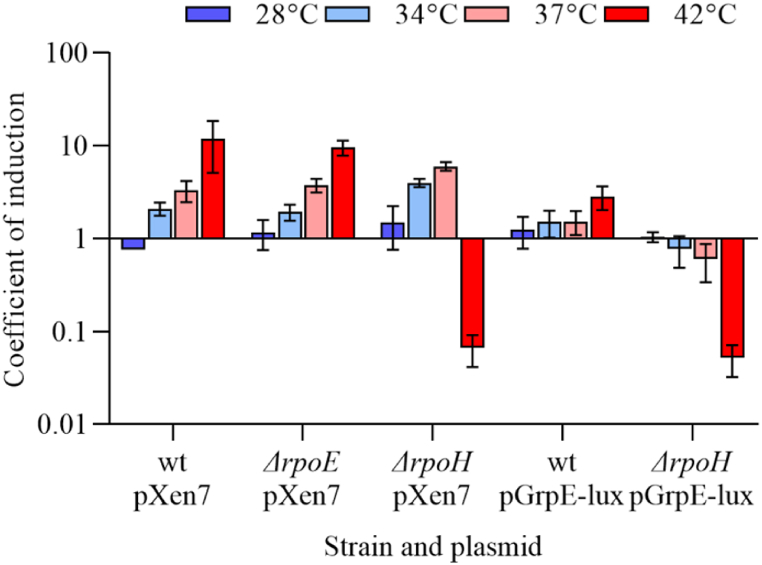
Induction coefficients of *E. coli* MG1655, K165 and MG1655 *ΔrpoE* with pXen7 and pGrpE-lux plasmids after 3 h at 28, 34, 37, and 42 °C. Means and standard deviations were determined in three independent experiments.

To investigate the effect of σ^32^ and σ^24^ subunits on the activation of luminescence gene synthesis at high temperatures in *E. coli* cells, strains K165 (σ^32^ mutant), MG1655 *ΔrpoE* (σ^24^ mutant) and wild-type strain MG1655 were transformed by pXen7 and pGrpE-lux plasmids. *E. coli* MG1655, K165 and MG1655 *ΔrpoE* cells with the pXen7 and pGrpE-lux plasmids were divided into equal samples and then were incubated at 23, 28, 34, 37, and 42 °C for 3 h. The coefficients of induction (2) are shown in Fig. 6.

As can be seen on the histogram (Fig. 6), the activation of *lux*-operon in *E. coli* cells with pGrpE-lux plasmid does not occur in the K165 strain compared to MG1655, which is understandable, since the promoter of the *GrpE* gene is σ^32^ dependent. At 42 °C, a drop is observed due to the sensitivity of the K165 strain to high temperatures.

In *E. coli* MG1655, K165 and MG1655 *ΔrpoE* cells cloned with the pXen7 plasmid, the unit luminescence at 23 and 28 °C remains almost at the same level, while an increase is observed at 34 °C, and with temperature rising, continues to grow to 42 °C. The drop in the luminescence level at 42 °C for K165, is explained by the inability of bacteria to live at high temperatures. The key result of this experiment is the preservation of the induction of the *P. luminescens lux*-operon promoter in *E. coli* cells with the deleted genes of σ^32^ and σ^24^ RNA polymerase subunits.

## 4 Discussion

In this paper, it is shown that the *lux*-operon of *P. luminescens* ZM1 in the heterologous system of *E. coli* and *lux*-operon of the closely related natural strain *P. temperata* are activated in a temperature-dependent manner. Activation of the synthesis of luminescence genes occurs when the temperature rises from 23 to 34 °C for the *P. temperata* strain and from 23 to 42 °C in the *E. coli* MG1655 pXen7 strain (Fig. 4, Fig. 6), and this activation is due to the *lux*-operon promoter (Fig. 2). Reliable activation of *lux*-genes expression with the addition of ethanol is observed only in *P. temperata* cells (Fig. 5). This effect can be explained by the fact that the heat shock response regulation systems of *E. coli* cell do not recognize the promoter of the *lux*-operon of *P. luminescens*, and the response to an increase in temperature is associated with another mechanism, for example, possible regulation at the translation level with the formation of RNA-thermometers [26,[36], [37], [38], [39]]. To test this hypothesis, experiments were conducted in *E. coli* strains mutated by the genes of σ-heat shock factors. As it turned out, the temperature-dependent activation of the *lux*-operon of *P. luminescens* persists in strains deficient in both the *rpoH* and *rpoE* genes (Fig. 6). Thus, we can assume the presence of a secondary structure of the *lux*-operon mRNA, which melts regardless of the presence of σ-factors of heat shock in both *E. coli* and *Photorhabdus* cells, which in turn can regulate the translation of *lux*-genes depending on temperature. However, the presence of activation of the expression of the *lux*-operon of *P. temperata* with the addition of ethanol indicates in favor of protein-mediated regulation, since ethanol does not induce melting of RNA pins. Probably, in natural strains of *Photorhabdus* genus, there is a combined regulation involving both mechanisms.

Further investigations of *Photorhabdus lux*-operon regulation mechanisms could form the basis for the construction of the novel biosensor for detection of specific stress conditions [40]. Also, determination of molecular mechanism of this regulation can bring a new instrument for biotechnological genetic engineering, since many microbiological processes in biotechnology are conducted at 20–40 °C.

## 5 Conclusion

Induction of transcription of *lux*-operon from bacteria of *Photorhabdus* genus in response to heat shock is shown. Expression of both *lux*-operon of the *P. luminescens* ZM1 strain in the heterologous system of *E. coli* and *lux*-operon of the newly isolated closely related strain *P. temperata* FV2201 is activated with rising temperature from 23 to 42 °C and 23–34 °C respectively. Only *P. temperata lux*-operon expression is induced by ethanol. Transcriptional heat shock *lux*-operon activation is demonstrated by RT-PCR from 28 to 34 °C. Heat-shock activation promoter *Photorhabdus lux*-operon is shown, and it is not depend on *E. coli* transcriptional factors σ^32^ and σ^E^.

## Author contribution statement

V.V. Fomin: Conceived and designed the experiments; Performed the experiments; Analyzed and interpreted the data; Wrote the paper.

S.V. Bazhenov: Analyzed and interpreted the data; Wrote the paper.

O.V. Kononchuk; V.O. Matveeva: Performed the experiments.

A.P. Zarubina; S.E. Spiridonov: Contributed reagents, materials, analysis tools or data.

I.V. Manukhov: Conceived and designed the experiments; Analyzed and interpreted the data; Wrote the paper.

## Funding statement

The investigation of mechanisms of luminescence regulation was supported by Ministry of Science and Higher Education of the RF, project FSMF-2022-0007 “Development of technology for rational and highly productive use of agro- and bioresources, their efficient processing and obtaining safe and highquality sources of food and non-food products”.

The search and characterization of luminescent bacteria in Moscow was supported by Russian Science Foundation [21-64-00018].

In this study the equipment of Applied Genetics Resource Facility of MIPT was used [075-15-2021-684].

## Data availability statement

All experimental data is presented in the article and Supplementary meterials. The datasets generated during and analyzed during the current study are available from the corresponding author on reasonable request.

## Additional information

No additional information is available for this paper.

## Declaration of interest’s statement

The authors declare no conflict of interest.
