# Supplementary material for: Photorhabdus lux-operon heat shock-like regulation
Source: Heliyon. 2023 Mar 11;9(3):e14527. doi: 10.1016/j.heliyon.2023.e14527 (PMC10025913; doi:10.1016/j.heliyon.2023.e14527)
Supplement: Multimedia component 1 [file mmc1.docx]

**Supplementary materials**

**Plasmid construction**

The *P. luminescens* promoter site was amplified from pXen7 by Xen7Pfor and Xen7Prev primers (Table S1) and inserted into the pDEW201 vector at the restriction sites *EcoR*I and *BamH*I. As a result, a plasmid named pXenP was constructed.

The insertion of the P*_lac_* promoter was obtained by annealing the PlacFor19 and PlacRev19 oligonucleotides (Table S1) on top of each other. The resulting double-stranded DNA fragment had sticky ends for cloning into the pDEW201 vector at the restriction sites *Bam*HI and *Kpn*I. The pDlac plasmid was obtained by ligation of the insert with the *Bam*HI/*Kpn*I linearized pDEW201 vector.

Table S1 - Primers used in the study

| Primer | Sequence |
| --- | --- |
| 16S_Ph_unidir | 5’-GGATAACCACTGGAAACGGTGGCTAAT-3’ |
| 16S_Ph_unirev | 5’-GACTTCATGGAGTCGAGTTGCAGA-3’ |
| Xen7Pfor | 5'-GAAGGAATTCAGGGGTCATCTTCTTCTA-3' |
| Xen7Prev | 5'-GTAGGATCCGTCTTATTAGCCATCCATTTAATGGGGT-3' |
| PlacFor19 | 5'‑GATCTTTACACTTTATGCTTCCGGCTCGTATGTTGTGTGGAATTGTGAGCGGATAACAAGTAC-3' |
| PlacRev19 | 5'‑TTGTTATCCGCTCACAATTCCACACAACATACGAGCCGGAAGCATAAAGTGTAAA-3' |
| Ph_DluxC | 5'-GGAAAAATGAAGAATATTCAAGACGCAGG-3' |
| Ph_RluxC | 5'-AAGAGCGCGTTATCGGATGATTAGGG-3' |
| 16SuniD | 5’-CGTGCCAGCAGCCGCGGTAATA-3’ |
| 16SuniRmidi | 5’-GGCCCCCGTCAATTCATTTGAGT-3’ |

Table S2 - Unit luminescence of *E. coli* MG1655 pXen7 and *P. temperata* FV2201 cells after incubation during 3 hours at 23, 28, and 34°C.

| **Number of replicate** | **Unit luminescence, RLU/OD** | | | | | |
| --- | --- | --- | --- | --- | --- | --- |
|  | ***E. coli* MG1655 pXen7** | | | ***P. temperata*** | | |
|  | **23°C** | **28°C** | **34°C** | **23°C** | **28°C** | **34°C** |
| 1 | 70833 | 54386 | 177174 | 1944 | 6364 | 44444 |
| 2 | 114667 | 88095 | 222222 | 10222 | 21086 | 73913 |
| 3 | 142222 | 109167 | 268750 | 5405 | 4762 | 8638 |
| 4 | - | 58823 | 476190 | 1542 | 9230 | 61250 |
| 5 | - | 103448 | 484848 | 2884 | 4666 | 70000 |
| 6 | - | 7778 | 26436 | - | 14634 | 60606 |
| 7 | - | - | - | - | 11666 | 205882 |
| 8 | - | - | - | - | 18627 | 136363 |
